# The Influence of Hearing Aid Type on Reading: Results of an Eye-Tracking Study at University

**DOI:** 10.3390/audiolres16020033

**Published:** 2026-02-27

**Authors:** Regina Fefelova, Ilia Poputnikov, Mikhail Mozgovoi, Mikhail Konstantinov

**Affiliations:** The Head Educational, Research and Methodological Center for Vocational Rehabilitation of Persons with Disabilities, Bauman Moscow State Technical University, 105005 Moscow, Russia; poputnikov@bmstu.ru (I.P.); mozgovoy@bmstu.ru (M.M.); mdk@bmstu.ru (M.K.)

**Keywords:** eye-tracking, reading, students with hearing impairments, hearing impairment, monaural hearing aids, binaural hearing aids, cognitive load, cognitive characteristics, educational materials

## Abstract

Background/Objectives: The study examines the characteristics of reading written texts depending on the type of hearing aid (monaural or binaural) and the individual hearing compensatory device used (cochlear implant or hearing aid) by students studying engineering fields of study in inclusive higher education. Methods: The identification of the students’ characteristics while reading was carried out using an eye-tracker. Results: The data obtained by eye-tracking technology indicate that there are no significant differences in the gaze point indicators when reading everyday text between students with binaural hearing aids and students without hearing impairments. At the same time, students with monaural cochlear implants showed different gaze point indicators when reading everyday text compared to the results of groups of students without hearing impairments and students with binaural hearing aids. Significant differences were found in indicators related to pupil diameter, in particular between the groups of students with monaural cochlear implants and students with binaural hearing aids. Conclusions: The results demonstrate the need to adapt written teaching materials not only to take into account the characteristics caused by the hearing impairment itself, but also to take into account individual characteristics caused by the type of hearing aid. However, given the small sample size (13–14 people in each group) and multiple variables included (types of devices and number), the results should be interpreted with caution and considered preliminary—additional studies involving a larger number of participants are needed to confirm the identified patterns.

## 1. Introduction

Effective teaching of students with hearing impairments is impossible without specially prepared educational conditions and materials tailored to their cognitive profile [1,2].

Depending on a number of factors, the cognitive profiles of students with hearing loss may vary, requiring an individual approach to educational needs in order to achieve learning objectives [2].

For individuals with monaural cochlear implants and binaural hearing aids, the perception and processing of textual learning material may differ due to varying experiences with hearing aids and sound lateralization [3].

The perception of sound differs between users of hearing aids and cochlear implants, as these devices rely on fundamentally different mechanisms for converting and encoding acoustic information. Modern hearing aids are acoustic amplification systems: they increase the sound signal level and apply wide dynamic range compression, frequency correction, and a number of digital processing algorithms (noise reduction, directionality, sometimes frequency reduction) to make the signal accessible for perception while preserving the function of the hair cells of the cochlea and residual hearing [4]. Cochlear implants work by converting sound into electrical signals, analyzing it by frequency bands, after which sequences of electrical impulses stimulate the fibers of the auditory nerve, bypassing the hair cells of the cochlea [5].

These differences in sound signal processing lead to different perceptual consequences. Cochlear implant users typically experience limitations in spectral resolution and accuracy in representing fine frequency structure, which affect pitch discrimination, timbre, and music perception, while rhythmic components are usually recognized better than pitch and timbre components [6,7]. There are also differences in the process of speech recognition against background noise: a reduction in the number of effectively distinguishable ‘spectral channels’ and the peculiarities of sound signal coding impair speech intelligibility in competing acoustic conditions, although the preservation of low-frequency residual hearing may provide an advantage in noise by adding low-frequency acoustic cues [8,9]. For hearing aid users, the quality of perception is largely determined by the degree of residual hearing and amplification/compression parameters, since processing is aimed at optimizing audibility and dynamic range while preserving the transmission of auditory information [4].

Differences in the accessibility and quality of auditory–verbal information can indirectly affect the development of reading skills through the development of language competences and the formation of phonological representations of words. Empirical studies show that early and full auditory–verbal exposure is associated with better oral speech and language outcomes in children with hearing loss [10,11], and language outcomes, in turn, are important predictors of subsequent academic outcomes, including literacy/reading in cochlear implant users [12,13]. It is emphasized that even with early implantation and progress in auditory perception, some children remain at risk of early literacy deficits (including components of phonological awareness), which requires targeted language support and the development of reading predictors [14,15]. Consequently, differences in speech perception and encoding when using hearing aids and cochlear implants should be considered one of the factors indirectly influencing reading through the trajectories of language development and phonological processing, along with visual–orthographic skills, quality of education, and communicative environment.

In addition to the differences caused by the principle of operation of hearing aids (hearing aids and cochlear implants), the number of hearing aids used and their combinations (monoaural, binaural, and bimodal hearing aids) have a significant impact on speech and learning skills. Binaural and bimodal provision of auditory information on average increases the effectiveness of auditory perception through binaural processing mechanisms—improved source localization, spatial differentiation of speech and noise, and, as a result, increased speech intelligibility in noise [16,17].

There are currently studies describing the advantages of bilateral stimulation, i.e., the use of two hearing aids at once, for the development of spatial hearing and speech perception in competitive acoustic conditions [17]. This is fundamentally important, since the deficit of auditory information in unilateral stimulation is associated with persistent difficulties in distinguishing sound information in noise and in situations requiring spatial signal discrimination, which may indirectly affect the accessibility of speech material in educational communication [18].

At the same time, research data describe the advantage of using cochlear implants in the formation of oral speech and language in children with severe and profound sensorineural hearing loss, when hearing aids do not provide sufficient audibility and signal quality [11].

At the same time, there are results indicating that with moderate to severe hearing loss, with the correct selection and regular use of two hearing aids, some children can achieve levels of speech and literacy comparable to the age norm. In particular, in a study comparing groups of children with hearing aids and cochlear implants, the differences in open speech recognition tests and pronunciation skills were minimal, while in a number of language and academic indicators (including reading), the group with hearing aids demonstrated results that were no worse, and in some cases higher results; the authors also noted that children with moderate to severe hearing loss can develop oral speech within the normal range [19].

Consequently, the advantage in speech development is determined not only by the type of device, but also by the degree of hearing loss, residual hearing, the timeliness of intervention, the quality of device adjustment, and auditory–verbal rehabilitation. Under comparable audiological conditions, binaural hearing aids in some cases can indeed provide sufficient volume and quality of auditory information for age-appropriate speech and reading, whereas in cases of more severe hearing loss, the probability of achieving comparable outcomes is higher when using a cochlear implant or a cochlear implant in combination with a hearing aid/cochlear implant [20].

Text-based learning materials are one of the main ways of conveying educational information in higher education programs and therefore need to be adapted to take into account the specific difficulties that students with different types of hearing aids are likely to encounter.

In view of the above, the following research hypothesis was formulated: students with different types of hearing aids (cochlear implants or hearing aids) and different modalities (monocular and binaural) have different indicators of gaze microdynamics when reading written texts.

The aim of the study was to identify significant indicators of gaze microdynamics during the reading of written texts by students with monaural cochlear hearing aids and binaural hearing aids.

Existing research confirms the need to study cognitive processes in order to develop effective learning strategies [21,22]. In this case, eye-tracking studies become sources of objective indicators of written information perception parameters for identifying universal and individual solutions and methodological practices in the educational process [23], taking into account the specific nature of reading among students with hearing impairments in general and with different types of hearing aids in particular [24].

The development of methods and materials for students with hearing impairments who use different types of hearing aids requires a detailed study of the nature of perception and the accompanying cognitive load in the process of reading written texts. It is possible to use indicators of gaze microdynamics [25], recorded using eye-tracking technology, as an indicator of these characteristics.

## 2. Materials and Methods

During the study, a survey was conducted among 75 students of bachelor’s degree programs in engineering, studying under adapted educational programs (for students with disabilities), containing questions about the degree of hearing loss, type of hearing aid, age of the first hearing aid use, and the presence of ophthalmological disorders.

The survey results revealed the following students with hearing impairments:

- 2 students with binaural cochlear implants,
- 3 students with binaural cochlear implant and hearing aid,
- 5 students with monaural hearing aids,
- 16 students with monaural cochlear implant,
- 49 students with binaural hearing aids.

Most students use binaural hearing aids, making it difficult to form comparable groups in this study of special populations. This limitation also prevents reliable investigation of differences in very small groups, such as those with binaural cochlear implants.

Nevertheless, for research using an eye-tracker, students from two large groups were selected. Among the 16 students with monaural cochlear implants, 2 students were not suitable due to visual characteristics that distorted eye-tracking and reading fixation. Among the 49 students with binaural hearing aids, 14 students were selected to create a comparison group matched to the students with monaural cochlear implants in terms of hearing loss, course, age, gender, and entrance exam scores.

In addition to the two groups of students with hearing impairments and different types of hearing aids, students without hearing impairments also participated in the study. They were comparable in terms of course, age, gender, and entrance exam scores to the students with hearing impairments in the two groups. Thus, participant groups were matched descriptively on key demographic variables; however, statistical tests of group equivalence were not performed.

Ultimately, students aged 18–28 from a technical university participated in the study using an eye-tracker. They were divided into three independent groups matched for age, gender, course of study, degree of hearing loss (except for the group without hearing impairment) and entrance exam scores: group with monaural cochlear hearing aids—14 students; group with binaural hearing aids—14 students; group of students without hearing impairments—13 students.

The group of students with one cochlear implant consisted of two first-year students, ten middle-year students, and two graduates, among whom eleven participants were deaf in both ears, one had fourth-degree loss in both ears, and two had fourth-degree loss in the left ear and deafness in the right ear.

The group of students with two hearing aids consisted of three first-year students, nine mid-year students, and two graduates, including eleven participants with deafness in both ears, one with fourth-degree hearing loss in both ears, and two with fourth-degree hearing loss in the left ear and deafness in the right ear.

The group of students without hearing impairments consisted of two first-year students, nine middle-year students, and two graduates.

The exclusion criteria for participants included ophthalmological or neurological conditions that affected the quality of the recording, making it impossible to correctly register the gaze (including those manifested in the inability to pass calibration/verification) [26]. The presence of combined (multiple) developmental disorders affecting cognitive text processing strategies was also considered critical [27].

All students are native Russian speakers, have sufficient command of the language to study higher education programs, and were admitted to the university based on the results of three examinations, including a written Russian language exam.

The Gazepoint GP3 (GP3SD v2) device from Canadian company Gazepoint (Vancouver, BC, Canada) was selected as the gaze recording equipment—a compact infrared eye-tracker with an open API. Key characteristics: sampling frequency 60 Hz, accuracy 0.5–1°; permissible head displacement ±15 cm in depth, 25 cm horizontally and 11 cm vertically [28]. A 23-inch monitor with a resolution of 1920 × 1080 pixels was used to display text, ensuring the same brightness and uniform lighting for all participants, sufficient for comfortable reading, and excluding infrared and/or pulsating light sources [29].

To accompany the instructions, the Google Live Transcribe automatic speech recognition module was used [30] with parallel text output to a separate screen. This approach ensured the necessary level of quality in the provision of instructions [31] and provided visual support for students with hearing impairments [32].

The stimulus material was selected taking into account the fact that reading and comprehension strategies depend significantly on the type of text. Narrative and expository texts were distinguished [33]. Existing studies also reveal the characteristics of cognitive work depending on whether the text belongs to one of these types [34], including in individuals with hearing impairments [35].

Two texts in Russian were used as stimuli: a narrative everyday text and an expository educational text.

The narrative everyday text shown in Figure S1 contained 76 words and was an excerpt from the news channel of the Department of Inclusive Education about a past event.

The expository educational text shown in Figure S2 contained 153 words, 1 system of equations, and 5 formulas, and was an excerpt from educational materials on the subject of “Analytical Geometry.”

The texts were adapted for the screen so that they occupied the entire available reading area: the lines were aligned in width, and Times New Roman font was used, as it is the most common font in technical and scientific circles. The font size and line spacing were increased to ensure that the screen was completely filled. For everyday text, the font size was 18 pt; double line spacing was chosen, which not only allowed the entire monitor to be filled with text, but also simplified the binding of gaze points (and corresponding characteristics) to the word layout for future research, thereby ensuring greater accuracy of binding in the context of the upcoming more in-depth analysis. For educational text, the font size was 14 pt; the line spacing was double for the same reasons of mapping. In addition, this contributed to the correct registration of the text scanning path by the test subjects, comfortable perception [36] and ease of working with the text for users of glasses. The line consisted of 60 characters [37].

The research procedure included the following stages:

1. Introductory message. A brief theoretical justification of the research objective and its significance for the development of adaptive educational technologies were displayed on the screen. For participants with hearing impairments, the text was accompanied by sign language interpretation and automatic speech recognition [32].

2. Personal instruction. The experimenter individually explained how to use the eye-tracker [9]; speech was automatically transcribed by Google Live Transcribe and displayed on an auxiliary screen.

3. Calibration. A standard 9-point calibration sequence was used [38].

4. Verification. A four-point verification grid with a permissible deviation of <1.5° was used. If the threshold was exceeded, recalibration was performed [38].

5. Text reading. The participant read the presented text at a comfortable pace to themselves, excluding the influence of vocalization on the microdynamics of gaze [25,39,40].

The participants read the text in the “Conducting an Experiment” mode of the Neurobureau software (version 1.0 build rev.1361) [41,42]. The “Experiment Analysis” mode allowed us to export data with the characteristics of the students’ gaze, namely, time stamps of measurement points (at a frequency of 60 Hz), screen coordinates of the gaze, and pupil diameter [38]. This primary information was further processed using methods proposed by the software developer [42], with the calculation of key indicators of visual behavior associated with cognitive load [25,26,38,42].

The I-Velocity Threshold (I-VT) algorithm, recommended for reading continuous text, was used to mark events [26,42]. The velocity threshold for saccade classification was 70°/s; continuous sequences of samples with a velocity below the threshold and a duration of ≥60 ms were marked as fixations, combing neighboring ones (angular distance between centers ≤0.5°) [26,42].

The temporal boundaries of reading were determined manually in order to exclude from the analysis both eye movements preceding reading (before the first fixation on the first word of the first line) and those related to re-reading (after fixation on the last word of the last line).

Thus, the choice of methods and thresholds is consistent with the practices described in works on applied eye-tracking in reading studies using Russian language material [42], as well as with the guidelines and capabilities of the Neurobureau software package [38,41].

Pupil diameter data were also preprocessed with basic guideline to provide insights on cognitive processes during reading [43]. Data points marked by the eye-tracking system with low validity scores were excluded, as were data points with too high a dilation speed. For determining the later ones, a robust filter based on MAD values was implemented, with N = 3.5. This value and the filter in general follow guidelines proposed by Kret M.E. and Sjak-Shie E.E. [43]. The resulting data point array was completed with interpolated ones in missing segments and smoothed with a low-frequency filter for analyzing pupil dynamics.

In order to detect the stable component of the pupillary response (tonic pupil diameter) [44], baseline correction was performed.

Thus, setting the baseline pupil diameter allowed us to exclude from the analysis the data of the initial physiological reaction of the pupil to the change in perceived stimuli (the transition from calibration to reading texts). It also made it possible to compare records between participants without the influence of personal physiological parameters [45].

The baseline pupil diameter was calculated as the average pupil dilation in the time interval of 250 to 500 ms at the beginning of reading [46]. The start of this baseline interval for each entry was selected as the point of the most pupil constriction after the text appeared on the screen, which may be considered an indicator of the adaptation of the pupils to the presented stimulus after calibration, i.e., after the stimulus changed. The interval continued as long as the pupil diameter remained relatively stable [47] but ended immediately before the onset of dilation, associated with cognitive processing in the reading process [46].

In the process of analyzing the obtained pupil data, markers of cognitive load were of particular interest. Thus, data processing had an additional step of calculating the points of maximal and minimal diameter, which were taken as these markers. It was taken into consideration that the selected maximum should be after the baseline interval, as there it more likely corresponds with cognitive processing, as was mentioned before. The last 10% of the total text was not taken into account while searching for this maximum for similar reasons, since before the end of reading, an increase in diameter could be registered, which most likely indicated general excitement associated with the completion of reading and not cognitive load [46]. After this, a minimum point was found, with the idea of it being after the maximum one, as a decreasing pupil diameter trajectory indicates the lowering of cognitive load [46].

Thus, the analysis of gaze microdynamics was carried out using various variations of fixation, saccade, and pupil indicators capable of revealing the peculiarities of text information perception in the process of reading texts (everyday and educational).

The main indicators of eye-tracking results considered in this study are:

- Total fixation time (seconds)—the total time of all recorded fixations,
- Average fixation duration (seconds)—the average time of a single fixation, obtained by dividing the total fixation time by the number of fixations,
- Number of fixations—the number of fixations recorded,
- Number of saccades—the number of recorded saccades,
- Total scan path length (angular degrees)—the total distance traveled by the gaze between fixation points.
- Average saccade length (angular degrees)—the average distance travelled by the gaze within a single saccade, obtained by dividing the total scan path by the number of saccades,
- Reading time—the total time spent reading the text,
- Pupil diameter (delta from baseline in millimeters)—pupil diameter recorded during reading, specified as delta from the selected baseline value in millimeters,
- Pupil diameter (delta from baseline in percent)—pupil diameter recorded during reading, indicated as delta from the selected baseline value in percent,
- Part of text read (percent)—the proportion of the total reading time up to the moment of recording a particular pupil diameter value,
- Time from reading start (seconds)—the time interval from the start of reading to the moment of registration of a particular pupil diameter value.

It should be noted that some of those indicators are connected. Thus, to prevent errors and misleads in the significance analysis, some of them should be considered primary, especially in questions of indicating cognitive load. The secondary ones still to be reported address specialties in the reading process of the studied groups of students.

Because total fixation time in our experiment was computed over the entire text, it primarily captures overall reading time and rereading/refixations, since total-time measures are defined as sums of fixations. Consequently, we treat mean fixation duration as the primary index of moment-to-moment processing effort at fixation points [48] while reporting total fixation time as a complementary aggregate measure.

The number of fixations and saccades are similar characteristics, as one goes after the other [49]. Choosing between the two, we consider the first one to be primary, as it also indicates how often a given participant had to stop to process information [50].

Average saccade length is closely connected to the number of saccades and total scan path length, as the last one is a production of the other two [51]. Yet, additionally, scan path length becomes a relative metric of retracts inside of the text [52], so we consider it a primary characteristic.

Among pupillometry characteristics, those that indicate points of effort, ease and tiring [53] in terms of working load are important. Thus, the part of the text read and time from the start of reading are considered primary indicators of mental effort, and pupil diameter is presented as a secondary indicator of mental effort [54]. That said, those characteristics are to be taken with caution, as pupil metrics tend to reflect a lot of different physiological processes [55].

## 3. Results

The raw data on gaze points and pupil diameters used in this article are provided in Tables S1–S3 of the Supplementary Materials for the group without hearing impairment, the group with binaural hearing aids and the group with monaural cochlear implants, respectively. The processed data on gaze points and pupil diameter are provided in Table S4.

The data obtained from each sample were compared in pairs using the Mann–Whitney criterion. However, given the relatively small sample size and the number of outcome measures, all statistical analyses should be considered exploratory. The *p*-values were not corrected for multiple comparisons and are reported to identify potential patterns rather than to support confirmatory inference.

Additionally, the study was not powered a priori to detect small to moderate effects, and the absence of significant group differences for some variables should not be interpreted as evidence of equivalence.

No independent measures of reading proficiency, vocabulary knowledge, or language dominance were collected; therefore, group differences may partially reflect unmeasured variability in reading skill rather than hearing device characteristics alone.

The data processing also revealed large standard deviations observed for some eye-tracking measures. It likely reflects substantial inter-individual variability, and these findings should be interpreted as preliminary, supporting further investigation.

### 3.1. Analysis of the Comparison of the Results of Eye-Tracking Indicators for Reading Texts by Groups with Monaural Cochlear and Binaural Hearing Aids

Analysis of the results obtained using the Mann–Whitney criterion revealed statistically significant differences in reading indicators both between groups with and without hearing impairments and between subgroups with hearing impairments by type of hearing aid, confirming the presence of distinctive features of perception in these subgroups not only of educational material but also of everyday vocabulary in the reading process. The data are shown in Table 1.

The results obtained and analyzed based on the average fixation duration (*p* = 0.024) indicate a difference in the processing of everyday information in narrative text among students with monaural cochlear implants, manifested by longer delays in the perception of lexemes on average with statistically non-significant reading times.

The statistical significance of the reading time indicator recorded between the maximum pupil diameter and its minimum value (*p* = 0.039) during reading indicates increased cognitive load in students with monaural cochlear implants when perceiving textual educational information compared to the group of students with binaural hearing aids, as pupil constriction occurs more slowly when performing complex tasks [44]. This conclusion is supported by the fact that students with monaural cochlear implants work more intensively with the words of the educational text: a greater number of saccades (*p* = 0.024) and fixations (*p* = 0.024) reflects the increased load and complexity of lexical–semantic processing [25,26,38,42]; an increase in the scan path length (*p* = 0.002) could only have occurred due to returns to already read text when working with the same text [38,42], i.e., there were regressive saccades—the longer the total scanning path, the more regressive saccades there were and the longer they were. This behavior indicates additional verification and integration of the word into the context of the sentence, allowing for partial compensation for the difficulties of information processing [42]. The above-mentioned students paused significantly more often on words, read them more slowly, and returned to the text to reread important information. Thus, we can say that these students have a greater need for contextual cues compared to students with binaural prostheses [25,42].

Aside from that, extremely large values of SD for average fixation duration relative to corresponding means of students with binaural hearing aids exclusively in educational text can be noticed. It seems to be a result of long fixations by some participants of this group, and further analysis should take place with more samples involved, as was discussed earlier.

Summarizing the comparison of the results of the two groups of students with different types of hearing aids, we can speak of a statistically confirmed difference in visual behavior and perception load in the context of working with written educational texts (in terms of cognitive load).

The identified differences and peculiarities of information perception in the reading process indicate the need to take them into account in the process of preparing and designing educational materials not only for the category of students with hearing impairments, but also for subcategories that differ in the type of hearing aid.

### 3.2. Analysis Comparing the Results of Eye-Tracking Reading of Texts by Groups with Monaural Cochlear Implants and Without Hearing Impairments

Analysis of the results using the Mann–Whitney criterion (Table 2) revealed statistically significant differences in the indicators of reading everyday and educational texts between the groups, allowing us to gain a better understanding of the reading characteristics of students with monaural cochlear implants.

A comparison of statistically significant results of the parameters of the process of reading everyday text by students with monaural cochlear implants reveals a general tendency to work intensively with words due to an increased number of saccades (*p* = 0.027) and fixations (*p* = 0.031), which in turn reflects an increased cognitive load in the reading process and lexical–semantic difficulties in processing the material [25,26,38,42]. In addition to the above features, reading everyday narrative information is characterized by a longer processing time for everyday text as a whole, in accordance with the level of significance of the text reading time indicator (*p* = 0.029) and individual lexemes of the text, in accordance with the indicators of average (*p* = 0.011) and total fixation time (*p* = 0.012).

Analysis of significant pupil diameter indicators when reading everyday text allows conclusions to be drawn about the increased cognitive load experienced by students with monaural cochlear implants compared to students without hearing impairments, as evidenced by a narrower pupil diameter in millimeters (*p* = 0.011) and as a percentage (*p* = 0.011) of the baseline and their slow constriction (*p* = 0.006) with longer reading times, during which constriction occurred (*p* = 0.029), with prolonged pupil constriction times (*p* = 0.006) [44,56,57].

A comparison of statistically significant results of the parameters of the process of reading educational texts reveals a general tendency towards intensive work with words due to an increased number of saccades (*p* = 0.044) and fixations (*p* = 0.044), which in turn reflects an increased cognitive load in the reading process and lexical–semantic difficulties in processing the material [25,26,38,42]. In addition, when reading educational texts, there are additional differences in the number of returns to the read text in terms of the total scan path length (*p* = 0.009). The described indicator of returns with a smaller volume of text read before reaching the minimum pupil diameter (*p* = 0.038) suggests that students with monaural cochlear implants experienced increased cognitive load when perceiving written educational information earlier than the group of hearing students [25,38,42].

Based on the results of comparing the reading performance of different texts by the above-mentioned groups, it can be recommended that when selecting educational materials for students with monaural cochlear implants, reference inserts should be used to minimize backtracking and, consequently, avoid the load associated with text orientation.

### 3.3. Analysis of the Comparison of the Results of Eye-Tracking Indicators for Reading Texts by Groups with Binaural Hearing Aids and Without Hearing Impairments

Analysis of the results using the Mann–Whitney criterion (Table 3) revealed statistically significant differences between the groups only when reading educational texts.

No statistical significance was found in the reading indicators of everyday narrative text in students with binaural hearing aids, which suggests that they have sufficiently developed compensatory functions for perceiving written everyday information.

However, when reading educational texts, students with binaural hearing aids showed statistically significant indicators of increased cognitive load compared to the group of students without hearing impairments.

This extremely large SD for average fixation time can be observed not only for the group with binaural hearing aids, but for students without hearing impairments too. It seems to be a result of long fixations for some participants in each group. This fact, in addition to the absence of such behavior in the group with monaural cochlear implants, can be a coincidence but also may reveal some patterns that make those two groups similar and differentiates students with binaural aids from those with monaural ones. It should be further investigated.

We see that students with binaural hearing aids reached the minimum pupil size (*p* = 0.008) at an earlier stage of reading with equal reading duration (judging by the lack of statistical significance of this indicator), which means that they experienced cognitive load faster, as further confirmed by the time of registration of the minimum pupil diameter being within seconds of reading the text (*p* = 0.012).

The load was revealed by the indicator of shorter reading time before pupil constriction [56,57], while the pupil diameter did not differ, and there was no additional work with terminological vocabulary and contextual orientation, which allows us to conclude that the perception of written educational text is more labor-intensive.

### 3.4. General Conclusions on the Reading of Different Types of Text by Students from Different Groups

When interpreting pupil response indicators, it is important to bear in mind that changes in pupil diameter reflect not only variations in cognitive load, but also a wide range of non-cognitive and contextual factors. These include, in particular, changes in lighting and stimulus brightness, fluctuations in overall arousal and emotional reactivity, as well as dynamics of engagement and fatigue during prolonged task performance [54,55,58,59]. In addition, decreased motivational engagement is accompanied by changes in both the tonic and phasic components of the pupillary response [53], and fluctuations in attention are systematically associated with changes in pupil tone as a marker of arousal level [60,61].

Taking into account the above factors, measures were taken in this study to minimize confounding influences: standardized presentation conditions were ensured, and generally accepted analytical control procedures were applied (normalization relative to the pre-interval baseline; artefact and blink filtering); metrics were used that allow separate analysis of the tonic and phasic components of the pupillary response, which is in line with current recommendations for the design and analysis of pupillometric experiments [54,55].

Taking into account the above factors and based on the data obtained and processed, we observe a statistically significant difference in visual behavior, presumably indicating the presence of cognitive load [25,42] in students with different types of hearing aids in the process of reading texts. In particular, the reading results of students with binaural hearing aids may indicate more developed compensatory abilities to overcome difficulties in perceiving not only educational information but also everyday information in written form.

The results presented in Table 1, Table 2 and Table 3 provide insight into strategies for working with everyday and academic written texts in the reading process, not only for those with hearing impairments, but also for those with different types of hearing aids. In particular, the results of students with binaural hearing aids are close to those of students without hearing impairments in terms of the number of fixations and saccades when perceiving everyday information in written form, while students with monaural cochlear implants have a statistically significant difference in these indicators.

Thus, we suggest that binaural hearing aids help to level out the difficulties in perceiving everyday information in written form, and we confirm the assumption about differences in the cognitive profiles of students with different types of hearing aids.

Because cochlear implants and hearing aids differ fundamentally in signal processing, the present comparison does not isolate the effects of binaural versus monaural stimulation and should not be interpreted as evidence for or against a binaural advantage per se.

## 4. Discussion

The results obtained in the study confirm the hypothesis of significant differences in information perception depending on the type of hearing aid in the context of cognitive load when reading written texts. In this section, we will discuss the results obtained, their consistency with existing theories, and directions for further research.

Based on the results obtained, we see a need not only to adapt written teaching materials, but also to form study groups of students with hearing impairments according to the type of hearing aid, taking into account monaural and binaural hearing aids within the framework of inclusive education. Such a measure will allow for the individualization of the educational process and provide groups of students with the necessary sets of special technical teaching aids and adapted teaching materials without excess or deficiency, adapting the educational process to the pace of perception, form of presentation, and other individual needs of students.

The identified differences in results between groups with different types of hearing aids in terms of reading time and reading load can be interpreted within the framework of the cognitive load model [62]. Presumably, binaural hearing aids, by providing interhemispheric asymmetry in the perception of sound information, contribute to a reduction in the cognitive load associated with compensating for hearing loss, freeing up resources for text processing. In this case, this explains the shorter fixation duration and reduced number of regressions, reflected in the total scanning path length parameter, in students with binaural hearing aids.

Binaural hearing aids stimulate symmetrical activation of the temporal lobes during auditory information perception, improving the processing and memorization of acoustic and semantic images of words, which subsequently affects their recognition when reading written text, facilitating work with written materials [56]. The above statement can be confirmed by the study, which found fewer fixations among students with binaural hearing aids compared to the group of students with monaural cochlear implants, raising the issue of the importance of binaural hearing aids [3] for accelerating the processing of everyday information in the process of solving everyday tasks.

The observed stability of indicators when the text (educational materials) becomes more complex can be considered within the framework of the dual coding hypothesis [63]. Binaural processing probably enhances the interaction between verbal and visual subsystems, which is especially important when perceiving syntactically complex constructions.

Based on the above, we can talk about the benefits of adapting existing higher education teaching methods and materials, taking into account the physiological characteristics and needs of students with hearing impairments, depending on the type of hearing aid.

The effectiveness of the proposed adaptations certainly requires verification and research with an increase in the sample size of participants, both in terms of numbers and the use of technical hearing correction devices. At the same time, further research suggests an increase in the number of text options with control over complexity and types of educational texts.

## 5. Conclusions

The results of the study, taking into account the cautionary interpretations due to the small sample size, may indicate a systematically higher cognitive load when reading among students with hearing impairments compared to the results of students without hearing impairments. At the same time, students with binaural hearing aids cope with reading educational and everyday text materials presumably with less time and difficulty with working with vocabulary, according to the results of the analysis of saccade indicators and the overall text scanning path, compared to the group of students with monaural hearing aids.

The results obtained justify the feasibility of conducting additional studies with larger sample sizes to confirm the results obtained and to test the effectiveness of the proposed options for adapting written educational materials for these groups in order to reduce the cognitive load experienced in the process of perceiving educational information in text form.

Presumably, the preliminary introduction of key vocabulary (glossaries on the topic, highlighting of terms, brief definitions before the text) will reduce the complexity of initial lexeme recognition and decrease the average duration of fixations on words that carry meaning [64,65]. Visual aids for contextual orientation (structural subheadings, marked lists, section ‘road signs,’ graphic markers of connections) simplify the return to places in the text that require rethinking, facilitating the integration of what has been read into the macrocontext [66,67].

## Figures and Tables

**Table 1:** Eye-tracking indicators when reading different types of text (M ± SD) by students with different types of hearing aids.

Indicator	*p*	Group with Monaural Cochlear Implants	Group with Binaural Hearing Aids
Narrative everyday text			
Total fixation time (seconds)	0.062	28.35 ± 9.81	20.98 ± 6.54
Average fixation duration (seconds)	0.024	0.22 ± 0.03	0.18 ± 0.04
Number of fixations	0.306	128.57 ± 30.99	113.93 ± 24.22
Number of saccades	0.306	128.14 ± 30.67	113.71 ± 24.11
Total scan path length (angular degrees)	0.427	556.49 ± 134.04	544.78 ± 177.05
Average saccade length (angular degrees)	0.734	4.44 ± 0.90	4.88 ± 1.49
Expository educational texts			
Total fixation time (seconds)	0.077	56.81 ± 15.73	43.31 ± 22.05
Average fixation duration (seconds)	0.839	0.23 ± 0.03	0.3 ± 0.29
Number of fixations	0.024	254.29 ± 83.57	176.5 ± 81.41
Number of saccades	0.024	254.07 ± 83.28	176.14 ± 81.46
Total scan path length (angular degrees)	0.002	834.70 ± 361.50	460.9 ± 255.46
Average saccade length (angular degrees)	0.125	3.28 ± 0.96	2.78 ± 1.09
Reading time elapsed from registering the maximal pupil diameter to the minimal one (seconds)	0.039	41.15 ± 19.42	26.64 ± 16.85

**Table 2:** Eye-tracking indicators when reading different types of text (M ± SD) by students with monaural cochlear implants and without hearing impairments.

Indicator	*p*	Group with Monaural Cochlear Implants	Group Without Hearing Impairments
Narrative everyday text			
Total fixation time (seconds)	0.012	28.35 ± 9.81	19.06 ± 7.25
Average fixation duration (seconds)	0.011	0.22 ± 0.03	0.18 ± 0.03
Number of fixations	0.031	128.57 ± 30.99	103.43 ± 25.75
Number of saccades	0.027	128.14 ± 30.67	103.14 ± 25.86
Total scan path length (angular degrees)	0.114	556.49 ± 134.04	472.38 ± 102.19
Average saccade length (angular degrees)	0.482	4.44 ± 0.90	4.78 ± 1.31
Reading time	0.029	34.34 ± 11.01	25 ± 7.6
Minimum pupil diameter registered after maximum pupil diameter (delta from baseline in millimeters)	0.01	−0.63 ± 0.3	−0.3 ± 0.35
Minimum pupil diameter registered after maximum pupil diameter (delta from baseline in percent)	0.003	−16.24 ± 8.15	−6.98 ± 7.97
Reading time elapsed from start to registering minimum pupil diameter (seconds)	0.006	28.15 ± 11.09	17.63 ± 6.15
Reading time elapsed from registering the maximal pupil diameter to the minimal one (seconds)	0.029	19.82 ± 8.99	12.67 ± 6.77
Expository educational texts			
Total fixation time (seconds)	0.227	56.81 ± 15.73	50.9 ± 25.76
Average fixation duration (seconds)	0.701	0.23 ± 0.03	0.35 ± 0.33
Number of fixations	0.044	254.29 ± 83.57	187.5 ± 79.98
Number of saccades	0.044	254.07 ± 83.28	187.29 ± 80.02
Total scan path length (angular degrees)	0.009	834.70 ± 361.50	489.8 ± 179.83
Average saccade length (angular degrees)	0.265	3.28 ± 0.96	2.91 ± 1.1
Part of text read before registered minimum pupil diameter (percentage)	0.038	68.94 ± 21.75	84.19 ± 11.24

**Table 3:** Eye-tracking indicators when reading different types of text (M ± SD) by students with binaural hearing aids and without hearing impairments.

Indicator	*p*	Group with Binaural Hearing Aids	Group Without Hearing Impairments
Narrative everyday text			
Total fixation time (seconds)	0.329	20.98 ± 6.54	19.06 ± 7.25
Average fixation duration (seconds)	0.964	0.18 ± 0.04	0.18 ± 0.03
Number of fixations	0.150	113.93 ± 24.22	103.43 ± 25.75
Number of saccades	0.164	113.71 ± 24.11	103.14 ± 25.86
Total scan path length (angular degrees)	0.511	544.78 ± 177.05	472.38 ± 102.19
Average saccade length (angular degrees)	0.511	4.88 ± 1.49	4.78 ± 1.31
Expository educational texts			
Total fixation time (seconds)	0.571	43.31 ± 22.05	50.9 ± 25.76
Average fixation duration (seconds)	0.541	0.3 ± 0.29	0.35 ± 0.33
Number of fixations	0.701	176.5 ± 81.41	187.5 ± 79.98
Number of saccades	0.701	176.14 ± 81.46	187.29 ± 80.02
Total scan path length (angular degrees)	0.603	460.9 ± 255.46	489.8 ± 179.83
Average saccade length (angular degrees)	0.701	2.78 ± 1.09	2.91 ± 1.1
Text part read from the moment of maximum pupil diameter to the moment of minimum pupil diameter (percentage)	0.008	49.27 ± 23	71.11 ± 11.57
Reading time elapsed from registering the maximal pupil diameter to the minimal one (seconds)	0.012	26.64 ± 16.85	44.41 ± 19.35

## Data Availability

The original contributions presented in this study are included in the Supplementary Material. Further inquiries can be directed to the corresponding author.

## Supplementary Materials

The following supporting information can be downloaded at: https://www.mdpi.com/article/10.3390/audiolres16020033/s1, Figure S1: Image with the narrative everyday text and its English translation; Figure S2: Image with expository educational text and its English translation; Table S1: The raw data on gaze points and pupil diameters for the group without hearing impairment; Table S2: The raw data on gaze points and pupil diameters for the group with binaural hearing aids; Table S3: The raw data on gaze points and pupil diameters for the group with monaural cochlear implants; Table S4: The processed data on gaze points and pupil diameters for all groups.
