# Supplementary material for: The Influence of Hearing Aid Type on Reading: Results of an Eye-Tracking Study at University
Source: Audiol Res. 2026 Feb 27;16(2):33. doi: 10.3390/audiolres16020033 (PMC13010651; doi:10.3390/audiolres16020033)
Supplement: Supplementary file 1 [file audiolres-16-00033-s001.zip › Figure S1.pdf]

11 сентября прошла встреча с работодателями и старт программы карьерного наставничества «Шаг в профессию» для студентов ГУИМЦ. Мероприятие собрало студентов всех курсов и направлений подготовки, которые заинтересованы в своем профессиональном развитии. На встрече было много старшекурсников и выпускников.

Стажеры прошлых сезонов программы рассказали, как программа помогла им не только развить профессиональные навыки, но и наладить связи с потенциальными работодателями. Карьерные наставники подробно рассказали о возможностях, которые предоставляет программа, и дали практические советы по успешному построению карьеры.

Narrative everyday text

On 11 September, a meeting with employers was held and the Step into the Profession career mentoring programme for GUIMC students was launched. The event brought together students from all courses and fields of study who are interested in their professional development. Many senior students and graduates attended the meeting.

Interns from previous seasons of the programme talked about how the programme helped them not only develop professional skills, but also establish connections with potential employers. Career mentors spoke in detail about the opportunities offered by the programme and gave practical advice on how to build a successful career.
