# Supplementary material for: The Influence of Hearing Aid Type on Reading: Results of an Eye-Tracking Study at University
Source: Audiol Res. 2026 Feb 27;16(2):33. doi: 10.3390/audiolres16020033 (PMC13010651; doi:10.3390/audiolres16020033)
Supplement: Supplementary file 1 [file audiolres-16-00033-s001.zip › Figure S2.pdf]

Алгоритм решения системы уравнений методом подстановки:

1) в одном из уравнений системы (более простом) выразить одну переменную через другие;

2) полученное выражение подставить в остальные уравнения вместо этой переменной;

3) затем точно так же выражаем и подставляем другую переменную и т.д., пока не получим уравнение с одной переменной;

4) после решения этого уравнения и нахождения значения (или значений) одной из переменных – последовательно возвращаемся к ранее выраженным переменным, подставляя найденные значения.

5) записываем ответ.

Пример 1. Решить систему уравнений $\begin{cases} 2x + 5y = 1, \\ x - 10y = 3. \end{cases}$

Решение. Из второго уравнения очень просто выразить x, т.к. коэффициент при этой переменной в этом уравнении равен одному:

$$x - 10y = 3 \Leftrightarrow x = 3 + 10y.$$

Теперь подставим то, что получилось вместо x в первое уравнение системы. Мы получили уравнение с одной неизвестной, которое очень просто решить:

$$2(3 + 10y) + 5y = 1 \Leftrightarrow 6 + 20y + 5y = 1 \Leftrightarrow 25y = -5 \Leftrightarrow y = -\frac{1}{5}.$$

А теперь вернемся к выражению для переменной x и подставим в него полученное значение y:

$$x = 3 + 10 \cdot \left(-\frac{1}{5}\right) = 3 - 2 = 1.$$

Итак,

Ответ: $x = 1; y = -\frac{1}{5}$.

Ответ, кстати, принято записывать как координаты, то есть в таком виде: $(x; y)$. В случае трех неизвестных: $(x; y; z)$ и так далее.

То есть ответ в нашем примере можно записать так:

Ответ: $\left(1; -\frac{1}{5}\right)$.

Expository educational texts

Algorithm for solving a system of equations by substitution:

1) in one of the equations of the system (the simpler one), express one variable in terms of the others;

2) substitute the resulting expression for this variable in the other equations;

3) then express and substitute another variable in the same way, and so on, until we obtain an equation with one variable;

4) after solving this equation and finding the value (or values) of one of the variables, we return to the previously expressed variables, substituting the found values.

5) Write down the answer.

Example 1. Solve the system of equations $\begin{cases} 2x + 5y = 1, \\ x - 10y = 3. \end{cases}$

Solution. From the second equation, it is very easy to express x , since the coefficient for this variable in this equation is equal to one:

$$x - 10y = 3 \Leftrightarrow x = 3 + 10y.$$

Now substitute what we got for x into the first equation of the system. We get an equation with one unknown, which is very easy to solve:

$$2(3 + 10y) + 5y = 1 \Leftrightarrow 6 + 20y + 5y = 1 \Leftrightarrow 25y = -5 \Leftrightarrow y = -\frac{1}{5}.$$

Now let's return to the expression for the variable x and substitute the obtained value y into it:

$$x = 3 + 10 \cdot \left(-\frac{1}{5}\right) = 3 - 2 = 1.$$

So,

Answer: $x = 1; y = -\frac{1}{5}$.

Incidentally, it is customary to write the answer as coordinates, i.e. in the following form: $(x; y)$. In the case of three unknowns: $(x; y; z)$ and so on.

That is, the answer in our example can be written as follows:

Answer: $\left(1; -\frac{1}{5}\right)$.
